# Improving the Classification Effectiveness of Intrusion Detection by Using Improved Conditional Variational AutoEncoder and Deep Neural Network

**DOI:** 10.3390/s19112528

**Published:** 2019-06-02

**Authors:** Yanqing Yang, Kangfeng Zheng, Chunhua Wu, Yixian Yang

**Affiliations:** 1 School of Cyberspace Security, Beijing University of Posts and Telecommunications, Beijing 100876, China; qing0991@163.com (Y.Y.); wuchunhua@bupt.edu.cn (C.W.); yxyang@bupt.edu.cn (Y.Y.); 2 College of Information Science and Engineering, Xinjiang University, Urumqi 830046, China; 3 Guizhou Provincial Key Laboratory of Public Big Data, Guizhou University, Guiyang 550025, China

**Keywords:** intrusion detection, variational inference, improved conditional variational autoencoder, generator network, deep neural network

## Abstract

Intrusion detection systems play an important role in preventing security threats and protecting networks from attacks. However, with the emergence of unknown attacks and imbalanced samples, traditional machine learning methods suffer from lower detection rates and higher false positive rates. We propose a novel intrusion detection model that combines an improved conditional variational AutoEncoder (ICVAE) with a deep neural network (DNN), namely ICVAE-DNN. ICVAE is used to learn and explore potential sparse representations between network data features and classes. The trained ICVAE decoder generates new attack samples according to the specified intrusion categories to balance the training data and increase the diversity of training samples, thereby improving the detection rate of the imbalanced attacks. The trained ICVAE encoder is not only used to automatically reduce data dimension, but also to initialize the weight of DNN hidden layers, so that DNN can easily achieve global optimization through back propagation and fine tuning. The NSL-KDD and UNSW-NB15 datasets are used to evaluate the performance of the ICVAE-DNN. The ICVAE-DNN is superior to the three well-known oversampling methods in data augmentation. Moreover, the ICVAE-DNN outperforms six well-known models in detection performance, and is more effective in detecting minority attacks and unknown attacks. In addition, the ICVAE-DNN also shows better overall accuracy, detection rate and false positive rate than the nine state-of-the-art intrusion detection methods.

## 1. Introduction

In recent years, with the rapid development of cloud computing, LoRa, NB-IoT, 5G communication and artificial intelligence technologies, the internet of things (IoT) technology has also ushered in a boom-like development, and hundreds of millions of devices are connected to the Internet of Things. However, because many IoT nodes collect and store large amounts of user privacy data, IoT systems have become an ideal target for cyber attackers, and attacks on the Internet of Things are increasing [1,2]. Gemalto’s IoT security report shows that more than half of companies still can’t find out whether they have suffered IoT vulnerability attacks. In addition, the report surveyed 950 IT and business decision makers and found that only 59% of companies encrypted all IoT-related data [3]. The popularity of IoT technology and the intelligence of devices have brought great convenience to people, but the use of new technologies and intelligent devices has also brought new security and privacy risks. For example, on 29 January 2018, the top three banks (ABN AMRO, ING Bank, Rabobank) in the Netherlands were attacked by distributed denial of service (DDoS), blocking access to websites and internet banking services [4]. In February 2018, the Pyeongchang Winter Olympics in South Korea suffered a cyber attack, which caused the live broadcast to be interrupted [5]. Therefore, maintaining the security of the IoT system is becoming the focus of successful deployment of the IoT network, and detecting intruders is an important step to ensure the security of the IoT network. Intrusion detection is one of several security mechanisms to manage security intrusions [6]. It monitors network traffic for abnormal or suspicious activity and issues alerts when such activity is discovered. Intrusion detection system (IDS) can be classified into host-based intrusion detection systems (HIDS) and network-based intrusion detection system (NIDS). In this paper, we study a network-based intrusion detection system. We studied the use of generation models and deep learning techniques to build intrusion detection classifiers to detect a great variety of attacks, such as DoS (denial of service), probe, U2R (user to root), R2L (remote to local), worms, shellcode, backdoor, reconnaissance, generic attacks, etc.

Many researchers have introduced more and more innovative approaches to detect intrusions in recent years, including anomaly detection methods, shallow learning methods, deep learning methods, and ensemble methods. The anomaly detection methods calculate the distribution of normal network data and define any data that deviates from the normal distribution as an anomaly, such as Bayesian models [7,8], the Cluster algorithms (K-Means, spectral clustering, DBSCAN, etc.) [7], self-organizing map (SOM) [9], Gaussian mixture model (GMM) [10], and one-class SVM [11]. Shallow learning methods use the selected features to build a classifier to detect intrusions, such as support vector machines (SVM) [12], decision tree (DT) [13], and k-nearest neighbor (KNN) [14]. Deep learning methods can automatically extract features and perform classification, such as AutoEncoder [15,16], deep neural network (DNN) [17], deep belief network (DBN) [18,19,20,21], and recurrent neural network (RNN) [22]. The last category uses various ensemble and hybrid techniques to improve detection performance, including bagging [23], boosting [24], stacking [25], and combined classifier methods [26].

Deep learning is a data representation and learning method based on machine learning, which has become a hot research topic. It can automatically extract high-level latent features without manual intervention [27]. Deep learning is widely applied in many fields of artificial intelligence, including speech processing, computer vision, natural language processing and so on. Moreover, deep learning has been applied to network security detection [28,29]. However, there are still many problems with intrusion detection systems. First, different types of network traffic in a real network environment are imbalanced, and network intrusion records are less than normal records. The classifier is biased towards the more frequently occurring records, which reduces the detection rate of minority attacks such as R2L and worms attacks. Second, because of the high dimension of network traffic, the feature selection method in many intrusion models is first considered as one of the pre-processing steps [30], such as principal component analysis (PCA) and chi-square feature selection. However, these feature selection methods rely heavily on manual feature extraction, mainly through experience and luck, and these algorithms are not effective enough. Third, due to the large network traffic and complex structure, the traditional classifier algorithm is difficult to achieve high detection rate. Fourth, the network operating environment and structure in the real world are changing, for example, the Internet of Things and cloud services are widely used and various new attacks are emerging. Since many unknown attacks do not appear in the training dataset, traditional intrusion detection methods usually perform poorly in detecting unknown attacks.

Taking into account the above factors, we propose a novel intrusion detection method called ICVAE-DNN, which combines improved conditional variational AutoEncoder (ICVAE) with DNN. The variational AutoEncoder (VAE) is an important generation model consisting of an encoder (a recognition network) and a decoder (a generator network) that use deep neural networks to characterize the distribution of data and latent variables, which was proposed by Kingma et al. [31] in 2013. VAE can generate samples, but it is not possible to generate some specific samples based on the labels. Therefore, CVAE was developed by Kingma et al. [32] in 2014. The CVAE is an extension of VAE [33]. It embeds a one-hot encoded label vector in the encoder and decoder, and converts unsupervised training mode into supervised training mode. CVAE not only automatically extracts high-level features and reduces the dimensions of network features, but also generate new attack samples of the specified categories. In order to initialize the weight of the DNN hidden layers using the CVAE encoder, we have improved CVAE by embedding intrusion tags only in the decoder, but not in the encoder, named ICVAE.

This paper has the following main contributions. First, we use ICVAE to learn the distribution of complex traffic and classes through supervised learning. The network parameters of ICVAE encoder are used to initialize the weight of DNN hidden layers. Second, latent variables with Gaussian noise and specified labels are fed into the trained ICVAE decoder (generating network) to generate specific new attack records, so as to balance the training data and increase the diversity of training samples, thus improving the detection rate of minority attacks and unknown attacks. Third, DNN is used to automatically extract high-level features, and adjust network weights by back propagation and fine-tuning to better address the classification problem of complex, large-scale and non-linear network traffic. Finally, the proposed model is evaluated on the NSL-KDD [34,35] and UNSW-NB15 [36,37,38] datasets. Compared with the well-known classification methods, the proposed model not only reaches better overall accuracy, recall, and false positive rate, but also achieves higher detection rate in minority attacks and unknown attacks.

The remainder of this paper is organized as follows. The related works are introduced in Section 2. Section 3 describes the ICVAE and DNN algorithms. Section 4 proposes a novel intrusion detection model and shows in detail how the model works. Section 5 demonstrates the experimental details and results. Finally, Section 6 provides some conclusions and further work.

## 2. Related Works

Although there are CVAE-related work in other fields, there is no report on the combination of ICVAE and DNN for intrusion detection. Kawachi et al. [39] employed a VAE for supervised anomaly detection. Sun et al. [40] used a VAE to learn sparse representations for anomaly detection. Chandy et al. [41] used VAE as a deep generation model to simulate network attack detection problems. Osada et al. [42] employed VAE as a semi-supervised learning for intrusion detection. They use VAE to detect intrusions, not CVAE. Lopez–Martin et al. [16] used conditional VAE (CVAE) to build an ID-CVAE classifier to perform classification and feature recovery. The ID-CVAE uses the reconstructed test data and the nearest neighbor method based on the Euclidean distance to classify the test samples. However, our proposed model not only generates data according to categories, but also uses DNN classifier to perform classification.

The deep learning method integrates high-level feature extraction and classification tasks, overcomes some limitations of shallow learning, and further promotes the progress of intrusion detection systems. Recently, deep learning models have been widely used in the field of intrusion detection. Stacked AutoEncoders are used to detect attacks in IEEE 802.11 networks with an overall accuracy of 98.60% [43]. Ma et al. [44] presented a hybrid method combining spectral clustering and deep neural networks to detect attacks with an overall accuracy of 72.64% on the NSL-KDD dataset. The gated recurrent unit recurrent neural network (GRU-RNN) was used to build an intrusion detection system in an software defined network (SDN) with an accuracy of 89% [45]. Shone et al. [15] employed a stacked non-symmetric AutoEncoder and random forest (RF) to detect attacks. Muna et al. [46] proposed an anomaly detection technique for internet industrial control systems (IICSs) based on the deep learning model, which used deep auto-encoder for feature extraction and deep feedforward neural network for classification. Tamer et al. [20] employed the restricted Boltzmann machine (RBM) to classify normal and abnormal network traffic. Imamverdiyev [18] used the multilayer deep Gaussian–Bernoulli RBM method to detect DoS attacks with an accuracy of 73.23% on the NSL-KDD dataset.

The above intrusion detection evaluation results are very encouraging, but these classification techniques still have detection defects, low detection rate for unknown attacks and high false positive rate for minority attacks. In order to overcome these classification problems, this paper uses ICVAE decoder to generate new attack samples according to the specified intrusion categories, thereby improving the detection rate of unknown attacks and minority attacks. ICVAE encoder automatically learns the potential representation of input data and reduces the dimensions of features. Furthermore, the ICVAE encoder is used to initialize the weight of DNN hidden layers. Finally, it is easier for DNN to achieve global optimization by back propagation and fine tuning network parameters.

## 3. Background

### 3.1. Variational AutoEncoder (VAE)

**Figure 1:**
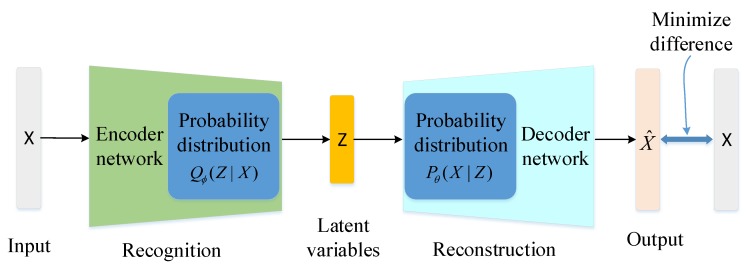
Variational AutoEncoder (VAE) architecture.

Variational AutoEncoder (VAE) is an important generation model consisting of an encoder network $Q_{\phi }\left(Z|X\right)$ and a decoder network $P_{\theta }\left(X|Z\right)$, as shown in Figure 1. VAE can learn approximate inference and can be trained using gradient descent method. The encoder network with parameters ϕ learns an efficient compression of the data into this lower-dimensional space, which maps data *X* into a continuous latent variable *Z*. The decoder network with parameters θ uses the latent variable to generate data, which maps *Z* to a reconstructed data $\hat{X}$. Here we use deep neural networks to construct the encoder and decoder with parameters θ and ϕ, respectively.

The core idea of VAE is to use the probability distribution P(X) to sample data points that match this distribution, where *X* represents a random variable of the data. The goal of VAE is to reconstruct the input data as much as possible, that is, to maximize the log likelihood probability of P(X) [31,47], as follows:

(1) $$\begin{array}{cc} \log P(X) & =E\left[\log P(X|Z)\right]-D_{KL}\left[Q\left(Z|X\right)|P\left(Z\right)\right]+D_{KL}\left[Q\left(Z|X\right)||P\left(Z|X\right)\right]\\ & \geq E\left[\log P\left(X|Z\right)\right]-D_{KL}\left[Q\left(Z|X\right)||P\left(Z\right)\right]. \end{array}$$

Here the variational lower bound objective [31,47] is defined as follows:

(2) $$L\left(\theta ,\phi ;X\right)=E\left[\log P\left(X|Z\right)\right]-D_{KL}\left[Q\left(Z|X\right)||P\left(Z\right)\right].$$

L is defined as the variation lower bound, which is called the VAE objective function. The first term in Equation (2) represents the reconstruction loss. It encourages the decoder to learn to reconstruct the input data. The second item in Equation (2) uses KL (Kullback–Leibler) divergence to minimize the difference between the encoder’s distribution Q(Z|X) and the prior distribution P(Z), that is to say, the learned distribution Q(Z|X) is similar to the prior distribution P(Z). Therefore, the goal of training VAE is to maximize the data generation probability logP(X|Z) and minimize the difference between the learned distribution Q(Z|X) and the true prior distribution P(Z). In other words, the goal of training VAE is to maximize the variational lower bound L.

### 3.2. Improved Conditional Variational AutoEncoder (ICAVE)

**Figure 2:**
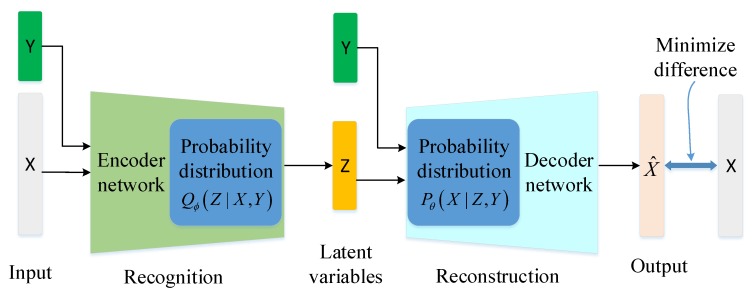
Conditional variational AutoEncoder (CVAE) architecture.

Conditional Variational AutoEncoder (CVAE) is an extension of VAE [33], modeled by conditioning the encoder and decoder to class *Y*, as shown in Figure 2. The encoder Q(Z|X,Y) is now conditional on two variables *X* and *Y*, and the decoder P(X|Z,Y) is now conditioned on two variables *Z* and *Y*.

Hence, the variational lower bound objective of CVAE [32,33] is defined as follows:

(3) $$\log P\left(X|Y\right)-D_{KL}\left[Q\left(Z|X,Y\right)∥P\left(Z|X,Y\right)\right]=E\left[\log P\left(X|Z,Y\right)\right]-D_{KL}\left[Q\left(Z|X,Y\right)∥P\left(Z|Y\right)\right]$$

**Figure 3:**
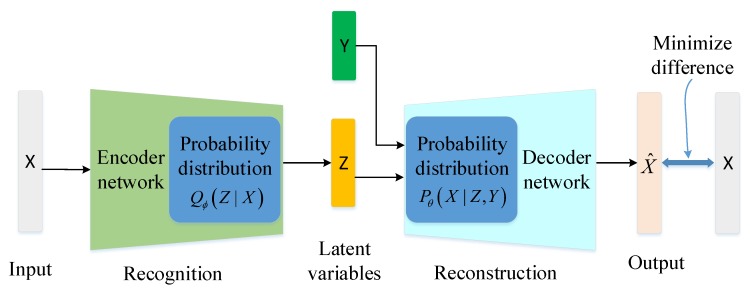
Improved conditional variational AutoEncoder (ICVAE) architecture.

The conditional probability distributions of CVAE encoder and decoder are related to class label *Y*. In order to use the encoder network of CVAE to initialize the network parameters of DNN, we improve the CVAE structure to embed class label *Y* only in the decoder network. The architecture of ICVAE is shown in Figure 3. The decoder is now conditioned to two variables *Z* and *Y* whereas the encoder is now conditioned to one variable *X*.

ICVAE is composed of encoder network $Q_{\phi }\left(Z|X\right)$ and decoder network $P_{\theta }\left(X|Z,Y\right)$. In the decoder, class labels are used as an extra input, so that the decoder probability distribution is conditional on the latent variable *Z* and class label *Y*, while the encoder does not contain the class label *Y*. When decoding, the latent variable *Z* and the label *Y* are connected and fed to the decoder, thus new attack samples of the specified class are generated. Hence, the variational lower bound of ICVAE is defined as follows:

(4) $$\log P\left(X|Y\right)-D_{KL}\left[Q\left(Z|X\right)∥P\left(Z|X,Y\right)\right]=E\left[\log P\left(X|Z,Y\right)\right]-D_{KL}\left[Q\left(Z|X\right)∥P\left(Z|Y\right)\right].$$

Here the variational lower bound objective of ICVAE is rewritten as:

(5) $$L\left(\theta ,\phi ;X,Y\right)=E\left[\log P\left(X|Z,Y\right)\right]-D_{KL}\left[Q\left(Z|X\right)∥P\left(Z|Y\right)\right].$$

$L\left(\theta ,\phi ;X,Y\right)$ in Equation (5) consists of two parts: a log reconstruction likelihood E[logP(X|Z,Y)] and a KL divergence $D_{KL}\left[Q\left(Z|X\right)∥P\left(Z|Y\right)\right]$. The first term is to reconstruct *X* by using the conditional probability distribution P(X|Z,Y) and the second term uses the KL divergence metric to characterize the encoder distribution Q(Z|X) approximating the prior distribution P(Z|Y). In ICVAE, we try to maximize the the variational lower bound objective $L\left(\theta ,\phi ;X,Y\right)$. In this model, We use the class label as our conditional variable *Y*. Obviously we could sample *Z* from a multivariate standard normal distribution N(0,I). By changing the value of *Y*, such as the attack class in the NSL-KDD dataset, ICVAE’s decoder P(X|Z,Y) can generate new attack samples of the specified category.

## 4. The Proposed Intrusion Detection Framework

**Figure 4:**
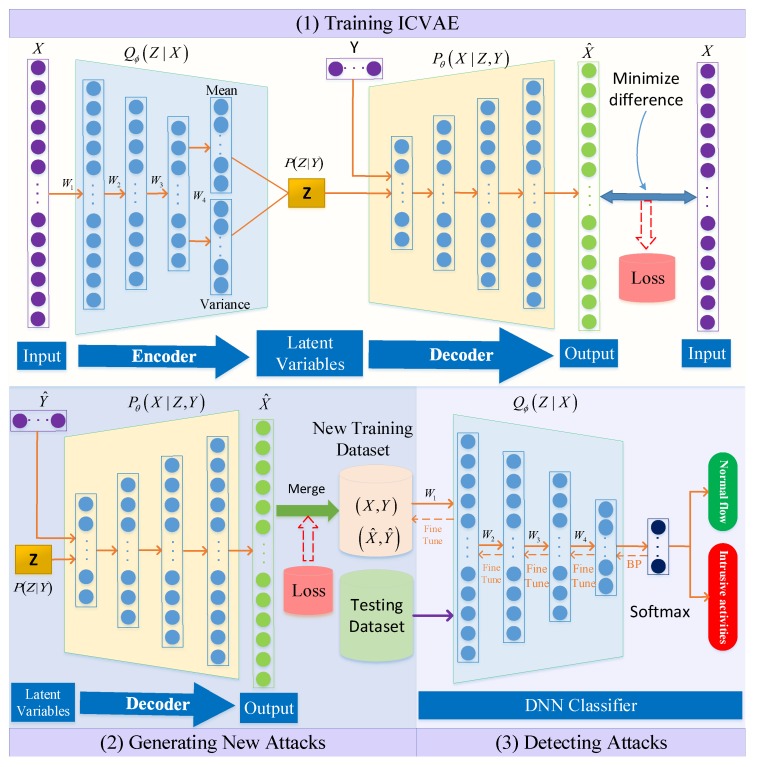
The proposed intrusion detection framework.

The framework of the proposed ICVAE-DNN is shown in Figure 4. ICVAE-DNN consists of three main phases: (1) training ICVAE, where the training samples are used to train the ICVAE, and the reconstruction loss for each training data sample is stored according to the attack class; (2) generating new attacks, where the ICVAE decoder generates new attack samples based on specified classes, and each newly generated attack sample is merged into the original training data set under the condition that the class reconstruction loss is satisfied; (3) detecting attacks, where the ICVAE decoder is used to initialize the weight of the DNN hidden layers, the merged training data set is used to train the DNN classifier, and the trained DNN classifier is used to detect attacks on the testing data set.

### 4.1. Training ICVAE

The input value of ICVAE must be a real vector, so each symbol feature in the intrusion detection dataset is first converted to a numerical feature. For example, the NSL-KDD [34,35] dataset contains 3 symbol features and 38 numerical features, and the UNSW-NB15 [36,37,38] dataset contains 3 symbol features and 39 numerical features. All symbol features are transformed to a binary one-hot encoding. The NSL-KDD and UNSW-NB15 datasets are converted into 122-dimensional and 196-dimensional features, respectively. The structure of ICVAE is composed of an encoder and a decoder, as shown in Figure 4. For the encoder Q(Z|X), we use a multivariate Gaussian distribution as the Q(Z|X) distribution. For the decoder P(X|Z,Y), we use a multivariate Bernoulli distribution to fit P(X|Z,Y). The output of the decoder network is reconstructed data, which is the predicted probability.

We use the min–max normalization method to scale all data *X* to [0,1]. After preprocessing all the data in the intrusion detection dataset, we train the ICVAE to optimize the loss of the encoder θ and the decoder ϕ by using the balanced sampling via label shuffling [48] and Adam [49] optimization algorithm. The ICVAE loss is composed of a reconstruction loss and a KL loss. The KL loss uses the variational inference method to approximate the distribution P(Z|Y) with the deep neural network Q(Z|X), so the ICVAE may have a KL-vanishing problem. ICVAE directly compares the difference between the reconstructed attack and the original attack through the encoding and decoding steps. However, the new attack samples generated by ICVAE decoder may differ greatly, and the newly generated samples may deviate from the original attack space distribution. In order to better select the newly generated attack samples, we calculate the reconstruction loss of each training sample based on the class and then use the maximum reconstruction loss for each class as the screening criteria.

We assume that the decoder $P\left(x_{i}|z,y\right),\left(where\;i=1,\cdots ,n\right)$ obeys the Bernoulli distribution, i.e.,

(6) $$\begin{array}{cc} P(x=1|z,y) & =\alpha_{z,y}, \end{array}$$

(7) $$\begin{array}{cc} P(x=0|z,y) & =1-\alpha_{z,y}. \end{array}$$

For an observation, the likelihood is:

(8) $$L=\alpha_{z,y}^{x}\cdot {\left(1-\alpha_{z,y}\right)}^{1-x}.$$

The decoder output is a parameter of Bernoulli distribution, that is, $\alpha_{z,y}=Decoder\left(z,y\right)=\hat{x}$. Then the negative log likelihood is:

(9) $$-\log L=-[x\cdot log\left(\hat{x}\right)+\left(1-x\right)\cdot \log\left(1-\hat{x}\right)].$$

It is obvious that the negative log likelihood in Equation (9) is the cross entropy. We use this cross entropy as the reconstruction loss of the decoder. After each training sample $\left(x_{i},y_{i}\right)$ is fed into the trained ICVAE, the reconstruction loss $l_{i}\left(x_{i},y_{i}\right)$ can be calculated as follows:

(10) $$l_{i}\left(x_{i},y_{i}\right)=-\left[x_{i}\cdot log\left(\hat{x_{i}}\right)+\left(1-x_{i}\right)\log\left(1-\hat{x_{i}}\right)\right].$$

The maximum reconstruction loss ${maxL}_{j}$ of the *j*-th class is written as follows:

(11) $${maxL}_{j}=k*\max\left\{l_{i}\left(x_{i},y_{i}\right)\right\},for\;\mathrm{each}\;y_{i}\in \mathrm{class}\;j\;,$$

where *k* represents the maximum reconstruction loss scaling factor, typically *k* is 1.0.

### 4.2. Generating New Attacks

For the encoder Q(Z|X), we use a multivariate Gaussian distribution as the Q(Z|X) distribution. For the decoder P(X|Z,Y), we define the multivariate standard normal distribution N(0,I) as the prior distribution P(Z|Y), that is, Z∼N(0,I). We can sample a latent variable *z* from N(0,I) under a specified label $\hat{y}$ and feed it into the trained decoder to generate a new attack sample $\left(\hat{x},\hat{y}\right)$. Assuming that the new attack sample belongs to class *j*, i.e., $\hat{y}\in \mathrm{class}\;j$, the generated sample $\left(\hat{x},\hat{y}\right)$ is fed into the trained ICVAE and the reconstruction loss $l(\hat{x},\hat{y})$ is calculated according to Equation (10). Then, we compare the reconstruction loss $l(\hat{x},\hat{y})$ with the maximum loss ${maxL}_{j}$ of the corresponding class *j*. If $l(\hat{x},\hat{y})$ < ${maxL}_{j}$, the newly generated sample is merged into the original training set *S*, otherwise the sample is discarded. The newly generated attack samples are merged into the original training set according to the following criteria:

(12) $$\mathrm{if}\;\hat{y}\in \mathrm{class}\;j\;\mathrm{and}\;l\left(\hat{x},\hat{y}\right)\leq {maxL}_{j},\;\mathrm{then}\;S=S\cup \left\{\hat{x},\hat{y}\right\},\;\mathrm{else}\;S=S.$$

### 4.3. Detecting Attacks

We employ DNN to detect attacks. DNN is a six-layer feedforward deep neural network. The activation function of all hidden layers in DNN is ReLU6 [50], and the activation function of the output layer in DNN is softmax. The network structure of DNN hidden layers is exactly the same as that of ICVAE encoder. ICVAE encoder can automatically extract high-level features, so the weight of the trained ICVAE encoder is used to initialize the weight of DNN hidden layers, then the merged training data set is used to fine tune DNN classifier, and the DNN classifier is optimized by Adam [49] algorithm. Finally, test samples are fed into the trained DNN classifier to detect attacks.

The proposed intrusion detection model is detailed in Algorithm 1.   

**Algorithm 1** Improved conditional variational AutoEncoder (ICVAE)-deep neural network (DNN).**Input:** Training dataset *S*, latent variable *Z*, learning rate lr, L2 regularization β and the maximum reconstruction loss scaling factor *k*.**Output:** the final classification results.
1:Data preprocessing: feature mapping and data normalization, all data is scaled to [0,1].2:The network structures of ICVAE on NSL-KDD and UNSW-NB15 datasets are 122-80-40-20-10-20-40-80-122 and 196-140-80-40-20-40-80-140-196, respectively. Weights are randomly initialized with scaling variance and biases are initialized to 0.3:Train the ICVAE using the training data set and calculate the maximum reconstruction loss maxL for each category in the training data set according to Equation (11).4:Sample *z* from the multivariate standard Normal N(0,I), specify the attack class $\hat{y}$, and feed them into the trained ICVAE decoder to generate a new attack sample $\hat{x}$. According to Equation (12), the newly generated sample $\left(\hat{x},\hat{y}\right)$ is merged into the training data set *S*.5:The weights of the trained ICVAE encoder are used to initialize the weight of the DNN hidden layers. First, all hidden layers are frozen, the parameters of output layer are adjusted by back propagation, then all hidden layers are unfrozen, and the merged training data set is used to fine tune DNN classifier.6:Test samples are fed into the trained DNN classifier to detect attacks.7:**return** the classification result.

## 5. Experimental Results and Analysis

### 5.1. Performance Evaluation

We use six commonly metrics to evaluate intrusion detection performance, including accuracy, detection rate (DR), precision, recall, false positive rate (FPR), and F1-score. Table 1 shows the confusion matrix consisting of true positive (TP), true negative (TN), false positive (FP), and false negative (FN). TP and TN indicate that the attack and normal records are correctly classified, respectively; FP represents a normal record that is incorrectly predicted as an attack; FN represents an attack record that is incorrectly classified as a normal record.

The accuracy, DR, precision, recall and FPR are defined as follows:

(13) $$\mathrm{Accuracy}=\frac{\mathrm{TP}+\mathrm{TN}}{\mathrm{TP}+\mathrm{TN}+\mathrm{FP}+\mathrm{FN}},$$

(14) $$\mathrm{DR}=\frac{\mathrm{TP}}{\mathrm{TP}+\mathrm{FN}},$$

(15) $$\mathrm{Precision}=\frac{\mathrm{TP}}{\mathrm{TP}+\mathrm{FP}},$$

(16) $$\mathrm{Recall}=\frac{\mathrm{TP}}{\mathrm{TP}+\mathrm{FN}},$$

(17) $$\mathrm{FPR}=\frac{\mathrm{FP}}{\mathrm{FP}+\mathrm{TN}}.$$

The F1-score is a measure of recall and precision using harmonic mean. Compared with the accuracy, F1-score is more suitable for evaluating the detection performance of imbalanced samples. It can be defined:

(18) $$F1-\mathrm{score}=\frac{2(\mathrm{Precision}*\mathrm{Recall})}{\mathrm{Precision}+\mathrm{Recall}}=\frac{2\mathrm{TP}}{2\mathrm{TP}+\mathrm{FP}+\mathrm{FN}}.$$

### 5.2. Datasets

Currently, the most common data sets used to evaluate the performance of network intrusion detection systems in the literature are the NSL-KDD [34,35] and UNSW-NB15 [36,37,38] data sets. Therefore, we selected the NSL-KDD and UNSW-NB15 data sets to validate the proposed model.

#### 5.2.1. NSL-KDD Dataset

The NSL-KDD is derived from the raw KDD Cup 99 [51,52] dataset presented by Tavallaee et al. [52]. The NSD-KDD dataset removes duplicate and redundant records in the KDD Cup 99 dataset and is more suitable for evaluating the performance of intrusion detection systems. There are five classes in the NSL-KDD data set, one normal and four attacks, namely, Probe, denial of service (DoS), user to root (U2R), and remote to local (R2L).

The NSL-KDD dataset is imbalanced, with fewer U2R and R2L records. We used two data sets in the NSL-KDD dataset to evaluate intrusion detection performance: KDDTrain+_20Percent.txt (A 20% subset of the full training set), KDDTest+.txt, and KDDTest-21.txt (A subset of the full test set, excluding records of difficulty level 21). In our experiments, KDDTrain+_20Percent is used as a training set, and KDDTest+ and KDDTest-21 are used as test sets. Table 2 shows the number of records for each category on the NSL-KDD dataset. As can be seen from Table 2, approximately 50% of the unknown attacks in the testing dataset did not appear in the training dataset.

#### 5.2.2. UNSW-NB15 Dataset

The UNSW-NB15 [36,37,38] is a new data set that reflects real modern normal activities and contains synthetic contemporary attacks. This data set is completely different from NSL-KDD, which reflects a more modern and complex threat environment. The raw network packet of the UNSW-NB15 data set was created by the Tcpdump tool, then 49 features with the class label are generated by Argus, Bro-IDS tool and 12 algorithms [38]. The full dataset contains a total of 25,400,443 records. The partition of the full dataset are divided into a training set and a test set according to the hierarchical sampling method, namely, UNSW_NB15_training-set.csv and UNSW_NB15_testing-set.csv. The training dataset consists of 175,341 records whereas the testing dataset contains 82,332 records. The number of features in the partitioned dataset [37] is different from the number of features in the full dataset [36]. The partitioned data set has only 43 features with the class label, removing 6 features (i.e., dstip, srcip, sport, dsport, Ltime and Stime) from the full dataset. The partitioned dataset contains ten categories, one normal and nine attacks, namely, generic, exploits, fuzzers, DoS, reconnaissance, analysis, backdoor, shellcode and worms. Table 3 shows in detail the class distribution of the UNSW-NB15 dataset.

### 5.3. Experimental Setup

Our experiments were carried out to evaluate the performance of the proposed model. We used three different datasets from NSL-KDD, and UNSW-NB15 datasets. We compared the results of the proposed model with other well-known detection methods. The proposed system was implemented in the TensorFlow environment on the ThinkStation with 64 GB RAM, Intel E5-2620 CPU and 64-bit Windows 10 operating system. An appropriate number of hidden layers can improve the generalization performance of the DNN classifier. Since the number of input units is less than 200, according to empirical experience, the candidate number of hidden layers is {3, 4}. DNN has the function of automatically extracting features, so the number of hidden units is set in a decreasing manner with the value of {two times the number of categories, four times the number of categories, eight times the number of categories, more than 8 times the number of categories}. When the learning rate is too large, the network will oscillate during training, resulting in no convergence. In TensorFlow, the default learning rate of the Adam optimizer is 0.001, which I reduced by 50%, so my candidate learning rate is {$1\times 10^{-3},5\times 10^{-4},1\times 10^{-4}$}. L2 is used to avoid over-fitting issue. Here, if L2 is zero then we get back the original model. However, if L2 is very large, it will add too much weight and will lead to under-fitting, so my candidate L2 is {$1\times 10^{-4},1\times 10^{-5}$}. In order to overcome the vanishing gradient problem caused by Sigmoid or the explosion gradient problem caused by ReLU, we consider ReLU6 [50] as the activation function of hidden Layers. The parameters of the ICVAE-DNN network configuration are searched according to the following principles, as follows:

- Number of hidden layers = {3,4}
- Number of hidden units = {two times the number of categories, four times the number of categories, eight times the number of categories, more than eight times the number of categories}
- Learning rate = {$1\times 10^{-3},5\times 10^{-4},1\times 10^{-4}$}
- L2 = {$1\times 10^{-4},1\times 10^{-5}$}
- Activation function of hidden Layers = {ReLU6}

**Figure 5:**
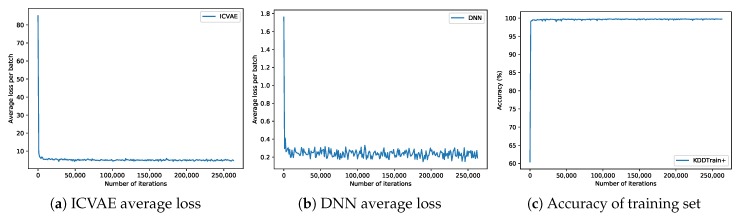
Training charts on the NSL-KDD dataset.

**Figure 6:**
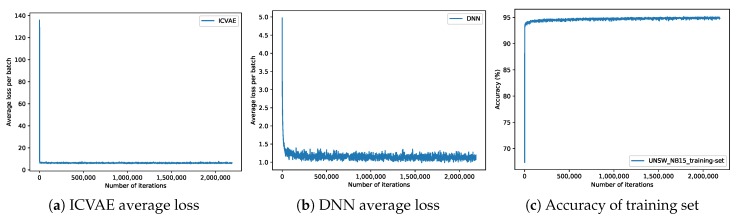
Training charts on the UNSW-NB15 dataset.

Grid search and three-fold cross-validation experiments are performed to find the optimal hyperparameters of a model which results in the most accurate predictions. The grid search traverses each group of hyperparameters in the search hyperparameter space. For each group of hyperparameters, three-fold cross-validation is used to evaluate. Three-fold cross-validation divides the original training dataset into three subsets, each of which shares the same proportion of each class of data. In each run of the model, two subsets are used to train the model and the remaining subset is used for test the model. By running the model three times, each subset of data has an equal chance to be used in testing part, and then the score of accuracy is computed by taking the average of the accuracy of the model on the testing subsets. Finally, the parameters that get the best cross-validation score are taken as the optimal parameter. The optimal network structures of the proposed model on the NSL-KDD and UNSW-NB15 data sets are 122-80-40-20-10-5 and 196-140-80-40-20-10, respectively. In the ICVAE encoder, the activation function of all hidden layers is ReLU6 [50], and the activation function of the output layer is linear. In the ICVAE decoder, the activation function of all hidden layers is ReLU6 [50], and the activation function of the output layer is Sigmoid. In the DNN, the activation function of all hidden layers is ReLU6 [50], and the activation function of the output layer is Softmax. The learning rate of ICVAE is $5\times 10^{-4}$, the learning rate of DNN is $1\times 10^{-4}$, the value of L2 regularization is $1\times 10^{-4}$, and the optimization algorithm is Adam [49]. Based on these optimal parameters, the training charts of ICVAE-DNN are shown in Figure 5 and Figure 6. As can be seen from Figure 5b and Figure 6b, the initial loss of DNN was relatively low, which implies that after initializing the weight of DNN hidden layers with ICVAE encoder, DNN was close to global optimum. From the average loss of ICVAE and DNN and the accuracy of the training data, it can be seen that the network is basically convergent.

We performed performance comparisons from two aspects: oversampling method and classification method. Table 4, Table 5, Table 6, Table 7 and Table 8 show the comparison results for different oversampling methods. Table 9, Table 10 and Table 11 show the performance comparison between the ICVAE-DNN and six well-known models. In addition, the detection performance of the ICVAE-DNN is further compared with other state-of-the-art models. Table 12 depicts the comparison results on the NSL-KDD (KDDTest+), NSL-KDD (KDDTest-21), and UNSW-NB15 datasets.

### 5.4. Results and Discussion

#### 5.4.1. The Detection Performance

As is evident from Table 2 and Table 3, the training samples are imbalanced on the NSL-KDD and UNSW-NB15 datasets. The U2R and R2L have minority records on the NSL-KDD dataset, and the worms and shellcode have minority records on the UNSW-NB15 dataset. We use ICVAE decoder to generate several records of the specified category to balance the training data, and the results are shown in Table 4 and Table 5.

The proposed ICVAE-DNN used the ICVAE decoder to synthesize minority attack samples. The most popular oversampling methods used to synthesize minority attack samples are random over sampler (ROS) [53], SMOTE [54], and ADASYN [55]. In order to demonstrate the superiority of ICVAE-DNN in oversampling technology, three classification models are constructed based on three oversampling methods, namely ROS-DNN, SMOTE-DNN and ADASYN-DNN. Table 6, Table 7 and Table 8 show the comparison results.

As can be seen from Table 6 and Table 7, the proposed ICVAE-DNN achieved the best detection performance on the NSL-KDD (KDDTest+) and NSL-KDD (KDDTest-21) data sets. Table 8 shows that the proposed ICVAE-DNN has a higher detection rate compared to all over-sampling methods except for ROS-DNN (only in backdoor attack), SMOTE-DNN (in DoS and reconnaissance attacks) and ADASYN-DNN (in fuzzers and analysis attacks) on the UNSW-NB15 dataset. ROS-DNN has a better detection rate in backdoor class compared to ICVAE-DNN (28.82% more), SMOTE-DNN shows a comparable detection rate to ICVAE-DNN in classes DoS and reconnaissance (14.73% and 3.52% more, respectively), and the detection rate of ADASYN-DNN in fuzzers and analysis attacks is 23.72% and 71.35% higher than that of our model, respectively. However, compared to three well-known oversampling methods, ICVAE-DNN has higher overall accuracy, precision, F1-score and FPR. These reasons may be due to defects in the three oversampling techniques of ROS, SMOTE and ADASYN. ROS-DNN is a simple copy of the training sample, which easily leads to model overfitting problems and reduces the generalization performance of the classifier. SMOTE-DNN uses the nearest neighbor method to generate new samples for each minority sample, which is prone to over-generalization. ADASYN-DNN uses the Γ distribution to automatically determine the number of samples that need to be synthesized for each minority sample, which are susceptible to outliers and cause changes in the spatial distribution of the original samples. ICVAE uses the spatial distribution of latent variables to generate samples under the guidance of class labels, and uses the reconstruction error to filter the generated samples to ensure that the generated samples are more consistent with the spatial distribution of the original samples. In addition, the trained ICVAE encoder was used to initialize the weight of the hidden layers of the DNN classifier, which made it easier for the DNN classifier to achieve global optimization, thereby improving classification performance. It was also demonstrated from Table 6, Table 7 and Table 8 that the proposed ICVAE is more suitable for solving the classification problem of imbalanced samples.

We compared the results of ICVAE-DNN with some well-known classification methods such as KNN (K-Nearest Neighbor), MultinomialNB (multinomial naive Bayes), SVM, RF, DNN, and DBN. We perform performance evaluation based on the five metrics introduced in Section 5.1. The results compared with some well-known classifiers are depicted in Table 9, Table 10 and Table 11.

As can be seen from Table 9, ICVAE-DNN had the highest overall accuracy, recall, precision and F1-score on NSL-KDD (KDDTest+) data set than all well-known classifiers, except RF (with 0.11% higher in FPR). Moreover, compared with other classifiers, ICVAE-DNN has a higher detection rate in DOS, U2R and R2L classes. RF has a slightly higher detection rate in the normal class compared to ICVAE-DNN (0.11% more), and MultinomialNB has a 7.64% higher detection rate in Probe class than ICVAE-DNN. However, ICVAE-DNN has the highest overall detection rate. In addition, ICVAE-DNN achieves the highest detection rates in two important minority U2R and R2L attacks, indicating that ICVAE-DNN is more effective in detecting minority attacks and unknown attacks.

Table 10 shows that ICVAE-DNN achieves the best overall performance, except for RF (only with 1.34% difference in the overall FPR). The detection rate of RF in the Normal class is 1.34% higher than that of ICVAE-DNN. In the probe attack, MultinomialNB achieved a 2.92% higher detection rate than ICVAE-DNN. However, RF and MultinomialNB were poor in other performances. ICVAE-DNN had the highest detection rates in minority classes U2R and R2L. Table 9 and Table 10 show that all classifiers have very low detection rates in the U2R and R2L attacks. The main reason was that there were too few U2R and R2L attacks in the training data set (with 11 and 209 samples, respectively). As can be seen from Table 2, almost half of the U2R and R2L attacks in the testing data set never appeared in the training data set, such as httptunnel, snmpgetattack, snmpguess, etc. As a result, all classifiers are not fully trained. Moreover, some attacks in the R2L class, such as sendmail and snmpguess attacks, exhibit features that were highly similar to normal records, which can cause the classifier to misclassify them as normal records.

Table 11 shows that ICVAE-DNN achieves the best overall performance compared to the other six well-known models, except for DBN in the overall recall (slightly higher by 3.22%). ICVAE-DNN reaches the highest detection rate in classes normal, reconnaissance, analysis, backdoor, shellcode and worms. MultinomialNB achieves the highest detection rate of 70.11% in the DoS class, which implies that the DoS attack features conform to the polynomial distribution. KNN achieved the highest detection rate of 96.63% in the generic class, but the overall DR of KNN was 1.67% lower than that of ICVAE-DNN. In fuzzers attacks, SVM achieved a 39.66% higher detection rate than ICVAE-DNN. The detection rate of DBN in exploits attack was 16.4% higher than that of our model, and the overall detection rate was also 3.22% higher, but its overall F1-score was 2.16% lower. However, ICVAE-DNN achieved the highest detection rates in minority and important attacks: analysis, backdoor, shellcode and worms. For example, the detection rate of ICVAE-DNN in the worms class was 68.44% and 79.55% higher than that of KNN and SVM, respectively. In addition, we can see that all classifiers have low detection rate in classes analysis and backdoor, mainly because the analysis and backdoor attack features are highly similar to exploits attack features. As a result, classifiers misclassify most of the analysis and backdoor attacks as exploits attacks.

### 5.5. Additional Comparison

To better demonstrate the performance of ICVAE-DNN, we compare its performance with nine state-of-the-art intrusion detection methods, namely, SCDNN (spectral clustering and deep neural network) [44], STL (self-taught learning) [56], DNN [57], Gaussian–Bernoulli RBM [18], RNN-IDS [58], CASCADE-ANN (a multiclass cascade of artificial neural network) [59], ID-CVAE (intrusion detection CVAE) [16], EM Clustering and DT [38]. Table 12 demonstrates the comparison results of ICVAE-DNN proposed on three datasets with other models in terms of accuracy, DR and FPR. Compared with other methods on the NSL-KDD (KDDTest+) and NSL-KDD (KDDTest-21) datasets, the proposed method achieved the best performance in terms of accuracy, DR and FPR. As shown in Table 12, ICVAE-DNN achieved the highest accuracy of 89.08% and DR of 95.68% on the UNSW-NB15 data set, but its FPR is slightly worse. The CASCADE-ANN proposed by Baig et al. [59] achieved a lower FPR (with 5.91% less) than ICVAE-DNN, but its accuracy and DR were worse. The experimental results show that ICVAE-DNN had higher accuracy, DR and FPR than other state-of-the-art intrusion detection methods except CASCADE-ANN (FPR on UNSW-NB15 dataset). Based on the experimental results, we concluded that ICVAE-DNN has better detection performance for network intrusion detection.

## 6. Conclusions

In this paper, we propose a novel intrusion detection approach called ICVAE-DNN that combines the ICVAE with DNN. For large data sets, ICVAE can learn and explore the potential sparse representations between network data features and categories. The trained ICVAE encoder is used to initialize the weight of DNN hidden layers. DNN can learn faster and easier than traditional multi-layer perceptron networks, thus avoiding stopping in the local minima. The ICVAE decoder is able to generate various unknown attack samples according to the specified intrusion categories, which not only balances the training data set, but also increases the diversity of training samples, so ICVAE-DNN can improve the detection rate of minority attacks and unknown attacks. DNN can automatically extract high-level abstract features from the training data, thus it can reduce data dimension to avoid dimension curse. DNN integrates feature extraction and classification methods into a system that automatically extracts features and performs classification without a lot of heuristic rules and manual experience. The classification performance of ICVAE-DNN is evaluated on the NSL-KDD (KDDTest+), NSL-KDD (KDDTest-21), and UNSW-NB15 datasets and compared with six well-known classifiers. Moreover, the experimental results show that the proposed ICVAE-DNN provides higher detection rates in minority attacks (i.e., U2R, R2L, shellcode and worms) than the six well-known classification algorithms: KNN, MultinomialNB, RF, SVM, DNN and DBN. In addition, compared with the state-of-the-art classifiers (such as SCDNN, STL, DNN, Gaussian–Bernoulli RBM, RNN-IDS, ID-CVAE, CASCADE-ANN, EM Clustering and DT), the proposed ICVAE-DNN achieves higher accuracy, detection rate and false positive rate. These experiments prove that ICVAE-DNN is more suitable for detecting network intrusion, especially for minority attacks and unknown attacks.

Considering future work, we plan to study an effective way to improve the detection performance of minority attacks and unknown attacks. We plan to use the adversarial learning method to explore the spatial distribution of ICVAE latent variables to better reconstruct input samples. Through the adversarial learning method, similar minority attacks can be synthesized, and the diversity of training samples can be increased. As a result, the detection performance of the ICVAE-DNN can be further improved.

## Figures and Tables

**Table 1:** The confusion matrix.

	Predicted Attack	Predicted Normal
Actual attack	TP	FN
Actual normal	FP	TN

**Table 2:** The class distribution of the NSL-KDD dataset.

0]*Category	Training Dataset		Testing Dataset			
	KDDTrain+_20Percent		KDDTest+		KDDTest-21	
	Attack	Count	Attack	Count	Attack	Count
Normal	normal	13,449	normal	9711	normal	2152
**Subtotal**		13,449		9711		2152
Probe	ipsweep	710	ipsweep	141	ipsweep	141
	satan	691	satan	735	satan	727
	portsweep	587	portsweep	157	portsweep	156
	nmap	301	nmap	73	nmap	73
			saint	319	saint	309
			mscan	996	mscan	996
**Subtotal**		2289		2421		2402
DoS	neptune	8282	neptune	4657	neptune	1579
	smurf	529	smurf	665	smurf	627
	back	196	back	359	back	359
	teardrop	188	teardrop	12	teardrop	12
	pod	38	pod	41	pod	41
	land	1	land	7	land	7
			apache2	737	apache2	737
			mailbomb	293	mailbomb	293
			processtable	685	processtable	685
			udpstorm	2	udpstorm	2
**Subtotal**		9234		7458		4342
U2R	buffer_overflow	6	buffer_overflow	20	buffer_overflow	20
	rootkit	4	rootkit	13	rootkit	13
	loadmodule	1	loadmodule	2	loadmodule	2
			perl	2	perl	2
			httptunnel	133	httptunnel	133
			ps	15	ps	15
			sqlattack	2	sqlattack	2
			xterm	13	xterm	13
**Subtotal**		11		200		200
R2L	guess_passwd	10	guess_passwd	1231	guess_passwd	1231
	warezmaster	7	warezmaster	944	warezmaster	944
	imap	5	imap	1	imap	1
	multihop	2	multihop	18	multihop	18
	phf	2	phf	2	phf	2
	ftp_write	1	ftp_write	3	ftp_write	3
	spy	1	named	17	named	17
	warezclient	181	sendmail	14	sendmail	14
			xlock	9	xlock	9
			xsnoop	4	xsnoop	4
			worm	2	worm	2
			snmpgetattack	178	snmpgetattack	178
			snmpguess	331	snmpguess	331
**Subtotal**		209		2754		2754
**Total**		25,192		22,544		11,850

**Table 3:** The class distribution of the UNSW-NB15 dataset.

Category	Training Dataset	Testing Dataset
	UNSW_NB15_Training-Set	UNSW_NB15_Testing-Set
Normal	56,000	37,000
Generic	40,000	18,871
Exploits	33,393	11,132
Fuzzers	18,184	6062
DoS	12,264	4089
Reconnaissance	10,491	3496
Analysis	2000	677
Backdoor	1746	583
Shellcode	1133	378
Worms	130	44
**Total**	175,341	82,332

**Table 4:** Number of samples generated on the NSL-KDD training dataset.

Category	Number of Original Records	Number of Newly Generated Records	Total
Normal	13,449	0	13,449
Probe	2289	11,160	13,449
DoS	9234	4215	13,449
U2R	11	13,438	13,449
R2L	209	13,240	13,449
**Total**	25,192	42,053	67,245

**Table 5:** Number of samples generated on the UNSW-NB15 training dataset.

Category	Number of Original Records	Number of Newly Generated Records	Total
Normal	56,000	0	56,000
Generic	40,000	16,000	56,000
Exploits	33,393	22,607	56,000
Fuzzers	18,184	37,816	56,000
DoS	12,264	43,736	56,000
Reconnaissance	10,491	45,509	56,000
Analysis	2000	54,000	56,000
Backdoor	1746	54,254	56,000
Shellcode	1133	54,867	56,000
Worms	130	55,870	56,000
**Total**	175,341	384,659	560,000

**Table 6:** Comparison of detection performance for different oversampling methods on the NSL-KDD (KDDTest+) data set (%).

Model	Normal	Probe	DoS	U2R	R2L	Accuracy	Recall	Precision	F1-Score	FPR
ROS-DNN	92.61	56.26	80.32	6.00	12.75	78.26	67.41	92.34	77.93	7.39
SMOTE-DNN	96.59	56.75	82.19	**11.00**	10.93	81.16	69.48	96.42	80.76	3.41
ADASYN-DNN	96.43	59.81	83.28	8.00	9.84	80.10	67.74	96.16	79.49	3.57
ICVAE-DNN	**97.26**	**74.97**	**85.65**	**11.00**	**44.41**	**85.97**	**77.43**	**97.39**	**86.27**	**2.74**

**Table 7:** Comparison of detection performance for different oversampling methods on the NSL-KDD (KDDTest-21) data set (%).

Model	Normal	Probe	DoS	U2R	R2L	Accuracy	Recall	Precision	F1-Score	FPR
ROS-DNN	85.83	65.36	74.14	5.50	10.02	63.43	58.46	94.89	72.35	14.17
SMOTE-DNN	86.76	60.99	66.86	12.00	14.45	65.34	60.59	95.37	74.10	13.24
ADASYN-DNN	67.98	54.29	67.94	8.00	11.58	57.76	55.50	88.65	68.26	32.02
**ICVAE-DNN**	**87.04**	**79.89**	**77.87**	**11.50**	**23.17**	**75.43**	**72.86**	**96.20**	**82.92**	**12.96**

**Table 8:** Comparison of detection performance for different oversampling methods on the UNSW-NB15 dataset (%).

Class	ROS-DNN	SMOTE-DNN	ADASYN-DNN	ICVAE-DNN
Normal	57.26	57.66	57.29	**80.99**
Generic	95.94	95.38	96.22	**96.31**
Exploits	49.69	50.59	44.36	**71.02**
Fuzzers	56.88	58.99	**59.07**	35.35
DoS	10.00	**22.65**	2.52	7.92
Reconnaissance	48.17	**83.81**	47.63	80.29
Analysis	13.44	15.36	**86.56**	15.21
Backdoor	**49.40**	42.20	0.86	20.58
Shellcode	90.74	84.39	80.69	**91.80**
Worms	34.09	52.27	47.73	**79.55**
**Accuracy**	80.52	80.92	80.72	**89.08**
**Recall (DR)**	99.50	**99.90**	99.85	95.68
**Precision**	74.04	74.30	74.12	**86.05**
**F1-score**	84.90	85.22	85.08	**90.61**
**FPR**	42.74	42.34	42.71	**19.01**

**Table 9:** Comparison of detection performance for different classification methods on the NSL-KDD (KDDTest+) dataset (%).

Model	Normal	Probe	DoS	U2R	R2L	Accuracy	Recall	Precision	F1-Score	FPR
KNN	92.78	59.4	82.25	3.50	3.56	76.51	64.19	92.16	75.68	7.22
MultinomialNB	96.03	**82.61**	37.1	0.50	22.22	78.73	65.64	95.62	77.85	3.97
RF	**97.37**	58.53	80.24	0.50	7.55	76.49	60.69	96.84	74.62	**2.63**
SVM	92.82	61.71	74.85	0.00	0.00	72.28	56.73	91.26	69.97	7.18
DNN	96.10	65.30	85.40	2.50	14.56	80.22	68.21	95.85	79.70	3.90
DBN	97.04	69.85	83.11	5.50	12.56	80.82	68.53	96.84	80.26	2.96
**ICVAE-DNN**	97.26	74.97	**85.65**	**11.00**	**44.41**	**85.97**	**77.43**	**97.39**	**86.27**	2.74

**Table 10:** Comparison of detection performance for different classification methods on the NSL-KDD (KDDTest-21) dataset (%).

Model	Normal	Probe	DoS	U2R	R2L	Accuracy	Recall	Precision	F1-Score	FPR
KNN	68.49	59.08	69.81	3.50	3.56	55.50	52.62	88.27	65.93	31.51
MultinomialNB	83.32	**82.81**	38.12	0.50	22.22	60.08	54.93	93.69	69.25	16.68
RF	**88.38**	60.45	66.08	0.50	10.42	56.84	49.84	95.08	65.39	**11.62**
SVM	68.26	61.41	56.79	0.00	0.00	47.38	42.74	85.85	57.07	31.74
DNN	86.29	67.86	64.30	4.50	13.94	60.96	55.34	94.79	69.88	13.71
DBN	71.75	58.33	71.72	0.50	13.25	57.45	54.28	89.65	67.62	28.25
**ICVAE-DNN**	87.04	79.89	**77.87**	**11.50**	**23.17**	**75.43**	**72.86**	**96.20**	**82.92**	12.96

**Table 11:** Comparison of detection performance for different classification methods on the UNSW-NB15 dataset (%).

Class	KNN	MultinomialNB	RF	SVM	DNN	DBN	ICVAE-DNN
Normal	74.56	57.78	76.42	57.64	74.31	69.68	**80.99**
Generic	**96.63**	96.29	96.73	96.24	96.41	96.34	96.31
Exploits	74.48	42.05	76.24	74.51	86.20	**87.42**	71.02
Fuzzers	42.33	42.48	53.33	**75.01**	45.53	55.10	35.35
DoS	19.44	**70.11**	10.37	0.00	7.65	8.24	7.92
Reconnaissance	58.94	36.76	78.52	0.57	77.46	79.81	**80.29**
Analysis	1.48	0.00	5.17	0.00	0.59	0.00	**15.21**
Backdoor	2.56	0.00	11.49	0.00	8.06	0.34	**20.58**
Shellcode	14.47	0.00	60.85	0.00	60.32	59.26	**91.80**
Worms	11.11	0.00	4.55	0.00	36.36	0.00	**79.55**
**Accuracy**	85.38	76.14	87.45	78.91	86.95	85.77	**89.08**
**Recall (DR)**	94.01	91.12	96.46	96.27	97.28	**98.90**	95.68
**Precision**	82.05	72.56	83.36	73.58	82.26	79.99	**86.05**
**F1-score**	87.63	80.79	89.44	83.41	89.14	88.45	**90.61**
**FPR**	25.19	42.22	23.58	42.36	25.69	30.32	**19.01**

**Table 12:** Comparison results based on NSL-KDD and UNSW-NB15 datasets (N/A means no available results, * Ranked first, ** Ranked second).

Method	Dataset	Accuracy (%)	DR (%)	FPR (%)
SCDNN [44]	NSL-KDD (KDDTest+)	72.64	57.48	N/A
STL [56]	NSL-KDD (KDDTest+)	74.38	62.99 **	7.21 **
DNN [57]	NSL-KDD (KDDTest+)	75.75	N/A	N/A
Gaussian–Bernoulli RBM [18]	NSL-KDD (KDDTest+)	73.23	N/A	N/A
RNN-IDS [58]	NSL-KDD (KDDTest+)	81.29 **	N/A	N/A
ID-CVAE [16]	NSL-KDD (KDDTest+)	80.10	N/A	N/A
**ICVAE-DNN**	NSL-KDD (KDDTest+)	85.97 *	77.43 *	2.74 *
SCDNN [44]	NSL-KDD (KDDTest-21)	44.55	37.85	N/A
STL [56]	NSL-KDD (KDDTest-21)	57.34	52.73 **	15.06 **
RNN-IDS [58]	NSL-KDD (KDDTest-21)	64.67 **	N/A	N/A
**ICVAE-DNN**	NSL-KDD (KDDTest-21)	75.43 *	72.86 *	12.96 *
CASCADE-ANN [59]	UNSW-NB15	86.40 **	86.74 **	13.10 *
EM Clustering [38]	UNSW-NB15	78.47	N/A	N/A
DT [38]	UNSW-NB15	85.56	N/A	N/A
**ICVAE-DNN**	UNSW-NB15	89.08 *	95.68 *	19.01 **
